# Intentional injury among the indigenous and total populations in British Columbia, Canada: trends over time and ecological analyses of risk

**DOI:** 10.1186/s12939-017-0629-4

**Published:** 2017-08-08

**Authors:** M. Anne George, Andrew Jin, Mariana Brussoni, Christopher E. Lalonde, Rod McCormick

**Affiliations:** 1 Department of Pediatrics, Faculty of Medicine, University of British Columbia, Vancouver, BC Canada; 2 BC Children’s Hospital Research Institute, Room F508, 4480 Oak Street, Vancouver, BC V6H 3V4 Canada; 3 School of Population and Public Health, University of British Columbia, Vancouver, BC Canada; 4 Epidemiology consultant, Surrey, Vancouver, BC Canada; 5 Department of Psychology, Faculty of Social Sciences, University of Victoria, Victoria, BC Canada; 6 Faculty of Human, Social and Educational Development, Thompson Rivers University, Kamloops, BC Canada

**Keywords:** Wounds and injuries, American Indian, Aboriginal, Indigenous population, Suicide, attempted, Hospitalization, Canada, Inequities

## Abstract

**Background:**

Our objective was to explore intentional injury disparity between Indigenous populations and the total population in the province of British Columbia (BC), Canada. We focus on hospitalizations, including both self-inflicted injuries and injuries inflicted by others.

**Methods:**

We used data from BC’s universal health care insurance plan, 1991 to 2010, linked to Vital Statistics databases. Indigenous people were identified through the insurance premium group, and birth and death records. Place of residence was identified through postal code. We calculated crude hospitalization incidence rates and the Standardized Relative Risk (SRR) of hospitalization, standardized by gender, 5-year age group, and Health Service Delivery Area (HSDA). With HSDA populations as the units of observation, linear regression was used to test hypothesized associations of Indigenous ethnicity, geographic, and socio-economic characteristics with SRR of injury.

**Results:**

During the period 1991–2010, the crude rate of hospitalization for intentional injuries was 8.4 per 10,000 person-years (95% confidence interval (CI): 8.3 to 8.5) for the total BC population, compared to 45.3 per 10,000 (95% CI: 44.5 to 46.1) for the Indigenous population. For both populations, risk declined over the period for injuries self-inflicted and inflicted by others. The linear regression model predicts that the off-reserve Indigenous population will have SRR of intentional injury 3.98 greater, and the on-reserve Indigenous population 4.17, greater than the total population. The final model was an excellent fit (R^2^ = 0.912, F = 177.632, *p* < 0.001), and found that three variables - occupational risk, high school diploma, and university degree – each provide independent effects when interacting multiplicatively with Indigenous ethnicity.

**Conclusions:**

The observation of substantially declining rates of intentional injury for both the Indigenous and total BC populations is off-set by the high disparity in risk between the two populations, which will likely continue until Canada reduces disparity with respect to discriminatory practices, and physical, social, and economic conditions.

## Background

Intentional injuries are either assaults inflicted by others or self-inflicted. With the aim to explore intentional injury risk among Indigenous populations in British Columbia (BC), Canada, we include both self-inflicted injuries and injuries inflicted by another, and all subsumed external cause categories, such as poisoning, drowning, firearms, cutting, falls, and attempted suicide.

Our previous work [1–12] has shown dramatic reduction in overall injury risk for both the total BC and Indigenous populations during the past two decades, including for children [6]. It has also shown decreasing, but persistent, disparity in risk between the two population groups, with more rapid rates of decreased disparity for some categories of injury (e.g., unintentional falls) [5, 10], compared to others (e.g., iatrogenic injuries) [11]. Our findings also highlight disparities in injury rates within Indigenous populations, with higher injury risk among rural and on-reserve communities [3, 8]. This report explores the category of intentional injury using the same population-based dataset with both total BC and Indigenous populations, and discusses similarities with and differences from risk of other categories of injury.

Canada recognizes three distinct Indigenous groups: First Nations, Inuit and Métis under the Constitution 1982 and Indian Act 1876 Section 35. The three groups are referred to as Indigenous for the purposes of this paper. In our team’s previous papers [1–12], we used the term Aboriginal peoples; however, this merely reflects a change in nomenclature. In BC, Indigenous peoples account for approximately 5% of the population.

Higher rates of intentional injuries have been reported amongst Indigenous, compared to non-Indigenous, populations in other colonized countries; for example, resulting from interpersonal violence in Australia [13] and for self-inflicted injuries in New Zealand [14]. In Canada, data have shown that Indigenous peoples are at higher risk than non-Indigenous populations of intentional injuries that result in either mortality [15–17] or hospitalization [18]. Oliver et al. [18] found that risk of self-inflicted injury was at least three times higher and assaults at least five times higher for those living in geographic areas with high, compared to low, concentrations of people who identify as Indigenous. In Alberta, Canada, self-inflicted injury rates were found to be highest among people supported by social assistance and for those with Indigenous status [19]. Thus, the epidemiological data represented by intentional injuries is important to comprehend because of the overall individual and societal burdens, and is particularly relevant for overrepresented populations, such as Indigenous peoples.

Considerable attention has been focused on the disproportionally higher rates of intentional injuries among Canadian Indigenous peoples and, in particular on violence against Indigenous women, in international reports [20, 21], national governmental [22–25] and non-governmental reports [26], and in the media [27]. A United Nations report noted that in Canada “Indigenous women and girls are also disproportionately victims of violent crime” [21]. The topic became a political issue during the 2015 federal election [28], and subsequently the newly elected government established a National Inquiry into Missing and Murdered Indigenous Women and Girls [29].

The present study extends and enhances our previous efforts to quantify the epidemiology of injuries among Indigenous peoples living both on- and off-reserve and in urban and rural places [2–12]. We use hospitalization data rather than mortality data because this is a much larger dataset since injury resulting in hospitalization is more common than injury resulting in death. We have improved methods for identification of the Indigenous population compared to previous studies in Canada (e.g., [18]) that use geography to identify the population, thereby making the assumption that all people living in specific places (e.g., reserves) are Indigenous. Instead, we have employed a method that identifies Indigenous people by record linkage to the provincial health insurance premium database and to Vital Statistics birth and death records.

The purposes of the current report are threefold: to compare intentional injury risk between the total and Indigenous populations of BC; to examine trends in risk by population group and sex over a 19-year time period; and to explore associations of risk with socioeconomic status, geographic place and ethnic identity. In addition, we discuss similarities with and differences from our results [5, 10–12] exploring risk of other injury categories.

## Methods

The University of British Columbia Behavioural Research Ethics Board reviewed and approved our methods (BREB file H06–80585). Data Stewards representing the BC Ministry of Health and the BC Vital Statistics Agency approved the data access requests. We used existing databases, permanently linked by British Columbia Personal Health Number, maintained by Population Data BC [30–33]. **Disclaimer**: All inferences, opinions, and conclusions drawn in this journal article are those of the authors, and do not reflect the opinions or policies of the Data Stewards.

We have published our methods in detail previously [2–11], including a discussion of the quality of the population registry, and validity and limitations of the Indigenous identification [5, 6] and provide a summary below.

### Population and hospital counts

As in previous analyses pertaining to other categories of injury [2–12], we used the premium billing files [30] of the province of BC’s universal health care insurance program, the Medical Services Plan of BC (MSP) as the population registry to calculate denominator populations for hospitalization rates. We classified persons as “Indigenous” according to method of insurance payment, which indicates the patient as having Indian Status, as defined by the Indian Act of Canada, or having Indian status noted on one’s own or on a parent’s linked Vital Statistics birth record [31] or death record [32]. This is an adaptation of a method previously developed and used by the Vital Statistics Agency of BC [34]. Within the Indigenous population, we classified people residing on an Indian Reserve or in an Indian Settlement or in an Indian Self-Governing District recognized by the federal government of Canada as “on-reserve”, and people not residing therein as “off-reserve”, according to their postal code of residence.

Hospital separations data [33] for residents of BC were available from April 1, 1991 through March 31, 2010. We considered a hospitalization as “due to injury” if the level of care was “acute” or “rehabilitation,” and the Most Responsible Diagnosis on the discharge record was an International Classification of Diseases Revision 9 (“ICD-9”) numeric code in the range 800 through 999, or an International Classification of Diseases Revision 10 (“ICD-10”) code in the range S00 through T98; and “intentional” if the first occurrence of the supplemental injury diagnosis code (indicating intention and external cause) was an ICD-9 E-code in the range E950-E958 or an ICD-10 code in the range X60-X84 (intentionally self-inflicted), or an ICD-9 E-code in the range E960-E968 or an ICD-10 code in the range X85-Y09 (purposely inflicted by another).

Linking hospitalization records to the population registry, we tabulated counts of hospitalizations by calendar year, gender, 5-year age group, Indigenous status, reserve residence, and residence within BC’s 16 Health Service Delivery Areas (HSDA) [34].

### Incidence rates of hospitalization

We calculated the crude rates of hospitalization per 10,000 person-years. We treated the crude rate as a binomial proportion and calculated 95% confidence limits accordingly. We calculated Standardized Relative Risk (SRR) of hospitalization, relative to the risk of hospitalization in the total population of BC during the same time period, using the method of indirect standardization [35], standardizing by gender, 5-year age group and HSDA in most cases when comparing population groups during the period 1991–2010, but standardizing by gender and 5-year age group when comparing HSDAs during the periods 1991–2010, 1999–2003 or 2004–2008. The SRR could also be called the Standardized Incidence Ratio.

We assessed cumulative change in SRR over time as the proportional change between the first and last years of the observation period, i.e., (SRR_2010_/SRR_1991_) −1. We converted change over the entire period to an annualized change, using this formula.

$$ {\left(\frac{SRR_{2010}}{SRR_{1991}}\right)}^{1/\left(2010-1991\right)}-1 $$

We compared the cumulative change (SRR_2010_ /SRR_1991_) among Indigenous people to the cumulative change among the total population of BC. We tested the statistical significance of the disparity (SRR_2010_ /SRR_1991_ Indigenous versus SRR_2010_ /SRR_1991_ BC) by calculating the probability (2-sided, *z*-test) that Ln((SRR_2010_)/(SRR_1991_)) Indigenous = Ln((SRR_2010_)/(SRR_1991_)) BC.

### Predictors of risk

Neither the population registry, nor the hospital discharge database, nor any other database linkable to these databases through Population Data BC, contained socioeconomic descriptors of individual clients. Therefore, we used an ecological approach to our analysis to examine risk markers, whereby the unit of observation was the HSDA (*n* = 16) subdivided into three population groups (total population, Indigenous off-reserve, and Indigenous on-reserve) and two time periods (1999–2003, and 2004–2008). Since two HSDAs had no Indian reserves, the total number of observation units was (14 × 3 + 2 × 2) × 2 = 92. The population units are not mutually exclusive (because the total population includes the two Indigenous subpopulations), therefore we did not use the group classification as a variable in the subsequent analysis. We did include the proportion of the population who are Indigenous as an analysis variable, because this an attribute of the observation unit, measured on a noncategoric scale.

Consistent with the ecological approach, we measured both outcome (i.e., injury risk) and predictors (i.e., hypothesized risk markers) at the level of HSDAs and population groups therein. Our hypothesized risk markers were socio-economic, housing, and geographic indicators previously developed by Statistics Canada and Indigenous and Northern Affairs Canada. From the Censuses of Canada, 2001 and 2006, we measured the following indicators, for the three population groups in each HSDA: (1) Total (annual) Income per capita, (2) the Income Score component (i.e., total annual income per capita, logarithmically scaled) of the Community Well-being Index [36], (3) proportion of population, age 25+ years with at least a high school certificate, (4) proportion of population, age 25+ years with university degree, bachelors or higher, (5) average population per room (an index of the degree of crowding in the population’s housing [37], (6) proportion of the population living in a dwelling in need of major repair, (7) proportion of population, age 25+ years, in the labour force, (8) proportion of population, age 25+ years, employed (for pay), (9) proportion of population who identified themselves as “an Aboriginal person, that is, North American Indian, Métis or Inuit (Eskimo)”, (10) proportion of population who gave only one response to the ethnic origin question, and it was a group that could be classified as North American Indian, (11) proportion of the HSDA’s population classified as “urban” (residing in a population centre with 100,000 or more persons), and (12) proportion of the HSDA’s population classified as “rural” (residing in a population centre with fewer than 1000 persons, or in an area with population density less than 400 persons per km^2^).

For each population group in each HSDA, we calculated the following work-related statistics of injury risk, relative to the population of BC: (13) relative risk of work injury compensation claim, expected from occupational categories, and (14) relative risk of work injury compensation claim, expected from industry categories. These two markers, defined in a previous report focusing on work-related injuries [2], describe the hazardousness of the distribution of the labour force among occupational and industrial categories. We also created four interaction terms, calculated as each of the employment-related risk markers multiplied by the proportion of the population who were employed, and by the proportion who were in the labour force. In regression analysis, the interaction terms model the effects of the hypothesized risk markers on risk of injury, with the effect varying according to the proportion of the population who are in the labour force, or who are employed. These may be interpreted as representing effects occurring specifically to the fraction of the population who are in the labour force, or who are employed.

Assuming that the effects of socioeconomic and geographic risk markers might be different for Indigenous peoples than for the general population, we created ethnicity interaction terms, calculated as each of the socioeconomic or geographic risk markers multiplied by the proportion of the population who were Indigenous. These interactions may be interpreted as representing effects occurring to the portion of the population who are Indigenous.

### Ecological analysis

For each HSDA sub-population, we calculated the age and gender standardized SRR of hospitalization due to intentional injury for two time periods, 1999 through 2003 (a 5-year period centred about the Census year 2001) and 2004 through 2008 (centred about the Census year 2006), relative to the total population of BC during the same time period. We used SRR as the dependent (Y) variable for regression analysis.

We tested hypotheses of association by performing least-squares linear regressions, weighted by person-years to diminish the impact of extreme values of SRR occurring in smaller population units. We tested census year, hypothesized socio-economic, work-related, geographic, and ethnicity markers, and interaction terms in turn as the single independent variable. Variables that had statistically significant association (*p* < 0.05) with SRR in univariate analysis were included in subsequent multivariable regression analysis. Beginning with the variable most strongly correlated with SRR (largest coefficient of determination R^2^ in the univariate analysis), we used stepwise forwards addition of variables to arrive at the best-fitting multivariable model. At each step, the variable with the largest *p*-value greater than 0.05 was eliminated. Addition and elimination stopped when all independent variables had regression coefficients significantly different from zero (*p* < 0.05) and the list of candidate variables was exhausted. In the final model, we tested the normality of the distribution of the standardized residuals by the Kolmogorov-Smirnov and Shapiro-Wilk statistics, and we verified homoscedasticity by scatter-plotting the standardized residuals against the regression-predicted values of SRR.

The regression coefficient (“B”) of each independent variable represents the mean change in the dependent variable SRR that is associated with unit change in the independent variable. The absolute change in SRR (i.e., SRR_2_ − SRR_1_) associated with a change of one standard deviation (SD) in the independent variable is calculated as*B* × *SD*.

We verified that the step-wise regression procedure (weighted by population) had indeed produced a model representative of the experience of the total population of BC and the much smaller Indigenous populations as well. We used the final regression model as a risk prediction calculator, then we compared the predicted disparities of injury SRR among the three population groups (total population, Indigenous off-reserve, and Indigenous on-reserve) to the observed disparities among the three groups; i.e., all HSDAs combined.

## Results

Table 1 shows observed and expected numbers of intentional injuries for the total BC population and Indigenous populations over the study period, 1991 to 2010. It also shows rates, SRR and their 95% confidence intervals (CI). Compared to the total BC population, the Indigenous population had more than threefold SRR for self-inflicted injuries and more than fourfold SRR for injuries inflicted by another person. Within the Indigenous population, the difference between those living on-reserve and those living off-reserve in SRR of self-inflicted injuries was not statistically significant (*p* = 0.284, two-sided). However, the SRR of injuries inflicted by another was higher among those living off-reserve (*p* = 0.001, two-sided).

**Table 1:** Hospital separations for intentional injuries^a^, British Columbia, 1991–2010^b^

	P-years^c^	Obs^d^	Exp^e^	Rate ^f^	95% CI for Rate			SRR^g^	95% CI for SRR		
BC, total population											
Intentional	78,256,306	65,802	65,802	8.4	8.3	to	8.5	1	[reference]		
Self-inflicted	78,256,306	38,590	38,590	4.9	4.9	to	5.0	1	[reference]		
Inflicted by another	78,256,306	27,212	27,212	3.5	3.4	to	3.5	1	[reference]		
BC, Indigenous											
Intentional	2,541,060	11,506	2990	45.3	44.5	to	46.1	3.85	3.71	to	3.99
Self-inflicted	2,541,060	6036	1738	23.8	23.2	to	24.4	3.47	3.31	to	3.64
Inflicted by another	2,541,060	5470	1252	21.5	21.0	to	22.1	4.37	4.13	to	4.62
BC, Indigenous, off-reserve											
Intentional	1,403,813	6009	1531	42.8	41.7	to	43.9	3.92	3.74	to	4.13
Self-inflicted	1,403,813	3029	889	21.6	20.8	to	22.4	3.41	3.19	to	3.64
Inflicted by another	1,403,813	2980	642	21.2	20.5	to	22.0	4.64	4.30	to	5.02
BC, Indigenous, on-reserve											
Intentional	1,131,862	5468	1457	48.3	47.0	to	49.6	3.75	3.57	to	3.96
Self-inflicted	1,131,862	2997	848	26.5	25.5	to	27.4	3.54	3.31	to	3.78
Inflicted by another	1,131,862	2471	610	21.8	21.0	to	22.7	4.05	3.74	to	4.39

^a^“Intentional injury” defined as Most Responsible Diagnosis in the range ICD9:800–999 or ICD10:S00-T98, and supplemental diagnosis in the range ICD9:E950-E958 or ICD10:X60-X84 (self-inflicted) or ICD9:E960-E968 or ICD10:X85-Y09 (inflicted by another)

^b^Injuries occurring during the observation period 1991-Apr-01 to 2010-Mar-31

^c^Person-years is the sum of the annual population counts times the fraction of each year included in the observation period

^d^Observed number of injuries

^e^Expected number, indirectly standardized, based on age, gender and HSDA-specific rates in the total population of BC

^f^Crude Rate per 10,000 person-years

^g^Standardized Relative Risk (compared to the total population of BC) = Observed/Expected

Table 2 shows gender and age-specific crude rates and SRRs for intentional injuries, among the total and Indigenous populations of BC during the period 1991–2010. Crude rates were highest in the 10–49 years age range, lower among children aged under 10 years and adults aged 50 years or older. Crude rates were higher among males than among females, in every age group except 10–19 years, where the rate was higher among females. These patterns are seen among both the total and Indigenous populations. Compared to the others of the same gender in the total population, and combining all age groups, intentional injury among Indigenous females (SRR = 3.98) was higher than among Indigenous males (SRR = 3.73, *p* = 0.019, two-sided).

**Table 2:** Hospital separations for intentional injuries^a^, British Columbia, 1991–2010^b^, by gender and age

		Total population						Indigenous population								
Gender	Age	Obs^c^	Rate^d^	95% CI for Rate			SRR [ref]	Obs^c^	Rate^d^	95% CI for Rate			SRR^e^	95% CI for SRR		
F	0–9	267	0.6	0.5	-	0.7	1	70	2.5	1.9	to	3.1	3.48	2.42	-	6.19
F	10–19	6427	13.0	12.7	-	13.4	1	1183	53.0	49.9	to	56.0	3.00	2.73	-	3.33
F	20–29	6536	12.2	11.9	-	12.5	1	1529	75.6	71.8	to	79.4	4.08	3.70	-	4.54
F	30–39	6706	10.9	10.7	-	11.2	1	1583	75.7	72.0	to	79.5	4.69	4.24	-	5.26
F	40–49	5223	8.4	8.2	-	8.6	1	839	49.6	46.3	to	53.0	4.33	3.80	-	5.04
F	50–59	2083	4.4	4.2	-	4.6	1	255	25.1	22.0	to	28.2	4.49	3.57	-	6.07
F	60–69	696	2.1	2.0	-	2.3	1	71	12.7	9.7	to	15.6	5.11	3.35	-	10.78
F	70–79	465	1.8	1.7	-	2.0	1	30	10.8	7.0	to	14.7	5.36	2.93	-	31.16
F	80+	348	2.0	1.8	-	2.2	1	6	4.2	0.8	to	7.5	2.20	1.01	-	NA
F	Total	28,751	7.3	7.2	-	7.4	1	5566	43.4	42.2	to	44.5	3.98	3.78	-	4.20
M	0–9	325	0.7	0.6	-	0.7	1	59	2.0	1.5	to	2.5	2.29	1.65	-	3.72
M	10–19	5223	10.0	9.8	-	10.3	1	809	34.8	32.4	to	37.2	2.86	2.56	-	3.24
M	20–29	10,994	20.8	20.4	-	21.1	1	2078	105.3	100.8	to	109.8	3.63	3.36	-	3.96
M	30–39	9146	15.2	14.9	-	15.5	1	1616	81.5	77.5	to	85.4	4.09	3.72	-	4.53
M	40–49	6753	10.9	10.7	-	11.2	1	930	59.9	56.1	to	63.7	4.29	3.78	-	4.95
M	50–59	2779	5.9	5.6	-	6.1	1	294	32.4	28.7	to	36.1	4.35	3.51	-	5.71
M	60–69	1024	3.1	2.9	-	3.3	1	112	23.0	18.8	to	27.3	5.69	3.94	-	10.18
M	70–79	523	2.4	2.2	-	2.6	1	33	14.7	9.7	to	19.8	4.62	2.67	-	17.33
M	80+	270	2.6	2.3	-	2.9	1	6	6.1	1.2	to	10.9	2.42	1.08	-	NA
M	Total	37,037	9.6	9.5	-	9.7	1	5937	47.5	46.3	to	48.7	3.73	3.56	-	3.93

^a^“Unintentional transportation injury” defined as hospital separation with Most Responsible Diagnosis in the range

ICD9:800–999 or ICD10:S00-T98, and supplemental diagnosis in the range ICD9:E800-E807, E810-E829, E831, E833-E838, E840-E848or ICD10:V01-V89, V91, V93-V99

^b^Injuries occurring during the observation period 1991-Apr-01 to 2010-Mar-31

^c^Observed number of injuries

^d^Crude Rate per 10,000 person-years

^e^Standardized Relative Risk (indirectly standardized by age, gender and HSDA, compared to the total population of BC) = Observed/Expected

**Fig. 1:**
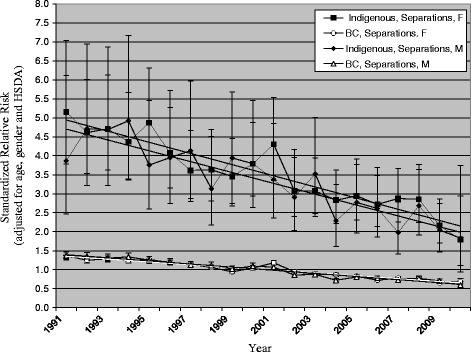
Injury hospitalizations in British Columbia, 1991–2010, relative risk by gender and year. Intentional Injury, Self-inflicted

Over the study period, risk decreased for self-inflicted injuries for both the Indigenous population and the total BC population, and for both sexes, as shown in Fig. 1. For the Indigenous population, SRR of self-inflicted injuries decreased from 3.87 to 1.83 for males (52.7% decrease), and from 5.15 to 1.79 for females (65.2% decrease). For the total BC population, SRR of self-inflicted injuries decreased for males from 1.37 to 0.60 (56.4% decrease), and for females from 1.37 to 0.68 (50.0% decrease). The decreases over time were not significantly different comparing Indigenous males to total BC males (*p* = 0.844), or comparing Indigenous females to total BC females (*p* = 0.224). Thus, considerable disparity remains between the total Indigenous and total BC populations.

**Fig. 2:**
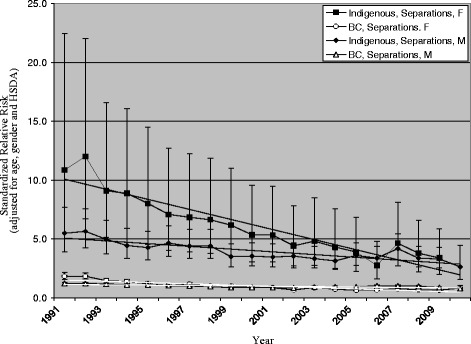
Injury hospitalizations in British Columbia, 1991–2010, relative risk by gender & year. Intentional Injury, Inflicted by another

For injuries inflicted by another person, similar downward trends for SRR were found, as shown in Fig. 2. For the Indigenous population, SRR decreased from 5.50 to 2.64 (51.9% decrease) for males, and from 10.86 to 5.76 (47.0% decrease) for females. For the total BC population, SRR for males decreased from 1.25 to 0.79 (36.5% decrease), and for females from 1.85 to 0.84 (54.5% decrease). Risk for Indigenous populations was considerably higher than risk for the total BC population in every year. As with self-inflicted injuries, the decreases were not significantly different comparing males in the two populations or females in the two populations, and considerable disparity remains between the total Indigenous and total BC populations.

Having established a consistently higher, although declining, risk for intentional injury for Indigenous peoples compared to the total BC population, we conducted ecological analyses to understand factors contributing to the disparity. Table 3 describes the three population groups (total BC population, Indigenous off-reserve population, and Indigenous on-reserve population), and their studied characteristics (intentional injury SRR, and socioeconomic, geographic, and ethnicity markers). In general, Indigenous populations have higher intentional injury risk, and are more socioeconomically disadvantaged and less urban than the total population. Similar tendencies are seen when comparing the on-reserve to the off-reserve Indigenous populations. However, the off-reserve Indigenous population has the highest labour force participation (higher than the total population), and the most hazardous employment (more than the on-reserve population).

**Table 3:** Descriptive profile of three population groups in British Columbia

Variable	Population group			
	Year	Total Population	Indigenous living off-reserve	Indigenous living on-reserve
Age and gender-SRR of hospital separation due to intentional injury	1999–2003	1	5.18	5.39
	2004–2008	1	4.94	5.28
Person-years of observation	1999–2003	20,663,214	363,704	301,529
	2004–2008	21,916,203	431,968	308,371
Mean annual person count	1999–2003	4,132,643	72,741	60,306
	2004–2008	4,383,241	86,394	61,674
Census total population	2001	3,868,875	123,640	46,385
	2006	4,074,380	145,020	51,060
Total Income per capita	2001	$22,890	$13,357	$9994
	2006	$27,370	$16,619	$10,797
Community Well-being Index Score	2001	81.4	63.4	53.7
	2006	87.3	70.7	56.3
Proportion of population, age 25+ years with at least a high school certificate	2001	0.720	0.590	0.496
	2006	0.834	0.716	0.530
Proportion of population, age 25+ years with university degree, bachelors or higher	2001	0.161	0.049	0.020
	2006	0.217	0.079	0.035
Average number of persons per room	2001	0.478	0.547	0.683
	2006	0.471	0.522	0.677
Proportion of population residing in dwelling requiring major repairs	2001	0.083	0.159	0.343
	2006	0.074	0.149	0.390
Proportion of population, age 25+ years, labour force participation	2001	0.658	0.677	0.641
	2006	0.658	0.701	0.616
Proportion of population, age 25+ years, employed	2001	0.611	0.549	0.470
	2006	0.624	0.626	0.476
Risk of work injury claim, relative to BC pop 2006, expected from occupation, labour force age 15+ years	2001	0.992	1.161	1.127
	2006	1.000	1.191	1.143
Risk of work injury claim, relative to BC pop 2006, expected from industry, labour force age 15+ years	2001	1.008	1.094	1.077
	2006	1.000	1.107	1.086
Proportion of population, Indigenous identity	2001	0.044	1.000	1.000
	2006	0.048	1.000	1.000
Proportion of population, North American Indian single response	2001	0.031	0.600	0.950
	2006	0.032	0.554	0.965
Proportion of HSDA population residing in large urban population centre	2001	0.608	0.375	0.216
	2006	0.616	0.371	0.216
Proportion of HSDA population residing in rural area	2001	0.145	0.231	0.292
	2006	0.142	0.232	0.290

Table 4 shows regression statistics from the preliminary regression models with one independent (X) variable, i.e., SRR = B*x* + Constant. The regression coefficient (B) and the “SRR change per SD” describe the association between the specified variable (*x*) and intentional injury risk (SRR). The coefficient of Determination (R^2^) measures the proportion of the variance in SRR explained by *x*. “P” is the probability of the null hypotheses that *R* ^*2*^ = 0 and B = 0 (i.e., no association between SRR and *x*).

**Table 4:** Ecologic analysis of risk of hospitalization due to intentional injury among Health Service Delivery Area population groups in British Columbia, 1999-2008^a^. Regression^b^ statistics from models with one independent (X) variable

X Variable	min	max	mean^c^	SD^c^	N	R^2^	B^d^	SE^e^	p^f^	SRR change per SD^g^	L95CL^h^	U95CL^i^
Census	2001	2006	2003.5	2.5	92	0.000	0.000	0.036	0.999	0.000	−0.181	0.181
Income Per Capita 1000	7.7	36.0	17.1	6.4	92	0.296	−0.111	0.018	0.000	−0.706	−0.934	−0.478
Income Score	45.1	96.5	69.5	12.4	92	0.421	−0.090	0.011	0.000	−1.110	−1.383	−0.837
High School	0.315	0.907	0.650	0.132	92	0.277	−5.558	0.946	0.000	−0.736	−0.985	−0.487
University Degree	0.000	0.364	0.084	0.076	92	0.211	−4.539	0.926	0.000	−0.344	−0.483	−0.204
Population Per Room	0.403	0.812	0.549	0.097	92	0.058	4.035	1.709	0.020	0.392	0.062	0.722
Need Major Repairs	0.050	0.478	0.186	0.116	92	0.616	16.377	1.364	0.000	1.901	1.586	2.216
Labour Force	0.515	0.771	0.664	0.053	92	0.001	−0.841	2.363	0.723	−0.045	−0.295	0.205
Employed	0.380	0.734	0.572	0.083	92	0.209	−9.191	1.888	0.000	−0.765	−1.077	−0.453
Occupation Risk	0.805	1.446	1.111	0.146	92	0.175	2.481	0.568	0.000	0.363	0.198	0.529
Industry Risk	0.687	1.258	1.064	0.108	92	0.134	2.911	0.780	0.000	0.315	0.147	0.482
Occupation Risk Employed	0.350	0.934	0.635	0.126	92	0.029	1.598	0.976	0.105	0.201	−0.043	0.444
Industry Risk Employed	0.299	0.826	0.609	0.113	92	0.003	0.610	1.256	0.628	0.069	−0.212	0.350
Occupation Risk Labour Force	0.510	1.055	0.739	0.124	92	0.144	3.145	0.809	0.000	0.389	0.190	0.588
Industry Risk Labour Force	0.448	0.900	0.708	0.102	92	0.098	3.324	1.062	0.002	0.339	0.124	0.553
Urban	0.000	1.000	0.386	0.416	92	0.189	−0.949	0.207	0.000	−0.394	−0.566	−0.223
Rural	0.000	0.446	0.228	0.153	92	0.191	2.642	0.574	0.000	0.404	0.230	0.578
Indigenous	0.007	1.010	0.676	0.447	92	0.832	4.521	0.214	0.000	2.022	1.832	2.213
North American Indian	0.004	0.992	0.501	0.377	92	0.810	5.655	0.289	0.000	2.132	1.916	2.348

^a^Three population groups (total, Indigenous on-reserve and Indigenous off-reserve) divided by 16 HSDAs and 2 time periods (1998–2003 and 2004–2008)

^b^The dependent (Y) variable is SRR of hospitalization due to intentional injury, and regression is weighted by person-years

^c^Unweighted mean and standard deviation (SD) of the independent (X) variable

^d^B = regression coefficient

^e^SE = standard error of the regression coefficient

^f^p = probability that B = 0

^g^SRR change per SD = BxSD. One SD change in the independent variable is associated with absolute change in the Standardized Relative Risk of injury by this amount. E.g., one SD change in Income Per Capita ($6400) is associated with change in SRR of −0.706 (decrease of 0.706)

^h^Lower limit of the 95% confidence interval for the SRR change per SD

^i^Upper limit of the 95% confidence interval for the SRR change per SD

Table 5 shows statistics from bivariate regression models where the independent term is a multiplicative interaction of Indigenous ethnicity with another variable (*x*), i.e., SRR = B*x*•*Ind* + Constant, where *Ind* is the proportion of the population who are Indigenous (i.e., 0 ≤ *Ind* ≤ 1). One may interpret this table as describing associations between each of the listed variables and falls injury risk in the Indigenous portion of the population (for whom *Ind* = 1).

**Table 5:** Ecologic analysis of risk of hospitalization due to intentional injury among Health Service Delivery Area population groups in British Columbia, 1999-2008^a^. Regression^b^ statistics from models with one independent (X) variable interacting with Indigenous ethnicity

X Variable	min	max	mean^c^	SD^c^	N	R^2^	B^d^	SE^e^	p^f^	SRR change per SD^g^	L95CL^h^	U95CL^i^
Census Indigenous	14	2026	1355	896	92	0.832	0.002	0.000	0.000	2.022	1.831	2.212
Income Per Capita 1000 Indigenous	0.2	22.0	9.2	6.2	92	0.758	0.313	0.019	0.000	1.942	1.712	2.172
Income Score Indigenous	0.6	80.1	42.5	28.0	92	0.811	0.071	0.004	0.000	1.992	1.791	2.193
High School Indigenous	0.005	0.871	0.405	0.279	92	0.748	7.119	0.435	0.000	1.985	1.744	2.226
University Degree Indigenous	0.000	0.149	0.032	0.032	92	0.458	54.398	6.234	0.000	1.747	1.350	2.145
Population Per Room Indigenous	0.004	0.812	0.398	0.279	92	0.822	7.402	0.363	0.000	2.063	1.862	2.264
Need Major Repairs Indigenous	0.000	0.478	0.158	0.143	92	0.713	15.093	1.008	0.000	2.155	1.869	2.440
Labour Force Indigenous	0.005	0.758	0.451	0.301	92	0.817	6.737	0.336	0.000	2.029	1.827	2.230
Employed Indigenous	0.004	0.714	0.373	0.254	92	0.772	8.021	0.459	0.000	2.039	1.807	2.271
Occupation Risk Indigenous	0.006	1.446	0.770	0.521	92	0.848	3.901	0.174	0.000	2.034	1.854	2.215
Industry Risk Indigenous	0.007	1.258	0.726	0.487	92	0.846	4.149	0.187	0.000	2.020	1.839	2.200
Occupation Risk Employed Indigenous	0.004	0.934	0.426	0.298	92	0.783	6.882	0.382	0.000	2.053	1.827	2.279
Industry Risk Employed Indigenous	0.004	0.826	0.402	0.280	92	0.782	7.314	0.407	0.000	2.049	1.822	2.276
Occupation Risk Labour Force Indigenous	0.004	1.055	0.514	0.353	92	0.828	5.771	0.277	0.000	2.038	1.843	2.232
Industry Risk Labour Force Indigenous	0.004	0.859	0.485	0.331	92	0.827	6.137	0.296	0.000	2.029	1.834	2.223
Urban Indigenous	0.000	1.004	0.253	0.378	92	0.122	3.408	0.963	0.001	1.289	0.565	2.012
Rural Indigenous	0.000	0.447	0.157	0.158	92	0.765	14.012	0.819	0.000	2.209	1.952	2.465

^a^Three population groups (total, Indigenous on-reserve and Indigenous off-reserve) divided by 16 HSDAs and 2 time periods (1998–2003 and 2004–2008)

^b^The dependent (Y) variable is SRR of hospitalization due to intentional injury, and regression is weighted by person-years

^c^Unweighted mean and standard deviation (SD) of the independent (X) variable

^d^B = regression coefficient

^e^SE = standard error of the regression coefficient

^f^p = probability that B = 0

^g^SRR change per SD = BxSD. One SD change in the independent variable is associated with absolute change in the Standardized Relative Risk of injury by this amount

^h^Lower limit of the 95% confidence interval for the SRR change per SD

^i^Upper limit of the 95% confidence interval for the SRR change per SD

Tables 4 and 5 show that almost all of the hypothesized predictors (both individually and as interactions with Indigenous ethnicity) are statistically significantly associated with injury risk (*p* < 0.05). However, because each model contains only one independent variable or term, the association may be due to confounding by another variable. We explored this further using multivariable models.

Table 6 shows the best-fitting multivariable regression model remaining after step-wise regression:

$$ \mathrm{SRR}={\mathrm{B}}_1{\mathrm{x}}_1\bullet \mathrm{Ind}+{\mathrm{B}}_2{\mathrm{x}}_2\bullet {\mathrm{x}}_3+{\mathrm{B}}_4{\mathrm{x}}_4+{\mathrm{B}}_5{\mathrm{x}}_5\bullet \mathrm{Ind}+{\mathrm{B}}_6{\mathrm{x}}_6\bullet \mathrm{Ind}+\mathrm{Constant}, $$

**Table 6:** Ecologic analysis of risk of hospitalization due to intentional injury among Health Service Delivery Area population groups in British Columbia, 1999-2008^a^. Regression^b^ statistics from best-fitting model with multiple independent (X) variables

X Variable	min	max	mean^c^	SD^c^	N	B^d^	SE^e^	p^f^	SRR change per SD^g^	L95CL^h^	U95CL^h^
(Constant)					92	2.160	0.444	0.000			
Occupation Risk Indigenous	0.006	1.446	0.770	0.521	92	7.598	0.780	0.000	3.962	3.154	4.770
Industry Risk LabourForce	0.448	0.900	0.708	0.102	92	1.436	0.390	0.000	0.146	0.067	0.225
Employed	0.380	0.734	0.572	0.083	92	−3.796	0.765	0.000	−0.316	−0.442	−0.189
High School Indigenous	0.005	0.871	0.405	0.279	92	−11.362	1.949	0.000	−3.168	−4.249	−2.087
University Degree Indigenous	0.000	0.149	0.032	0.032	92	38.779	7.082	0.000	1.246	0.793	1.698

Multivariable model statistics: R^2^ = 0.912, F = 177.632, *p* <0.001

^a^Three population groups (total, Indigenous on-reserve and Indigenous off-reserve) divided by 16 HSDAs and 2 time periods (1998–2003 and 2004–2008)

^b^The dependent (Y) variable is SRR of hospitalization due to intentional injury, and regression is weighted by person-years

^c^Unweighted mean and standard deviation (SD) of the independent (X) variable

^d^B = regression coefficient

^e^SE = standard error of the regression coefficient

^f^p = probability that B = 0

^g^SRR change per SD = BxSD. One SD change in the independent variable is associated with absolute change in the Standardized Relative Risk of injury by this amount

^h^95% confidence limits of the SRR change per SD

Where:

$$ \mathrm{Ind}=\mathrm{proportion}\  \mathrm{of}\  \mathrm{population}\ \mathrm{who}\ \mathrm{are}\ \mathrm{Indigenous}, $$

$$ {\times}_1=\mathrm{hazardousness}\  \mathrm{of}\  \mathrm{occupational}\  \mathrm{category}, $$

$$ {\times}_2=\mathrm{hazardousness}\  \mathrm{of}\  \mathrm{industry}\  \mathrm{category}, $$

$$ {\times}_3=\mathrm{proportion}\  \mathrm{of}\  \mathrm{population},\mathrm{age}\ 25+\mathrm{years},\mathrm{in}\  \mathrm{labour}\  \mathrm{force}, $$

$$ {\times}_4=\mathrm{proportion}\  \mathrm{of}\  \mathrm{population},\mathrm{age}\ 25+\mathrm{years},\mathrm{employed}, $$

$$ {\times}_5=\mathrm{proportion}\  \mathrm{of}\  \mathrm{population},\mathrm{age}\ 25+\mathrm{years},\mathrm{with}\  \mathrm{high}\  \mathrm{school}\  \mathrm{diploma}, $$

$$ {\times}_6=\mathrm{proportion}\  \mathrm{of}\  \mathrm{population},\mathrm{age}\ 25+\mathrm{years},\mathrm{with}\  \mathrm{university}\  \mathrm{degree}. $$

The final model was an excellent fit (R^2^ = 0.912, F = 177.632, *p* < 0.001). Standardized residuals were approximately normally distributed: Kolmogorov-Smirnov statistic was 0.101 (*p* = 0.022) and Shapiro-Wilk statistic was 0.971 (*p* = 0.039). Scatter-plotting of standardized residuals against the predicted values of SRR showed symmetrical distribution above and below, all along the horizontal baseline.

Two employment-related terms had no interaction with Indigenous identity; that is, these applied to Indigenous and non-Indigenous people alike. These were: being employed decreased the risk of intentional injuries, and the interaction between industry risk and labour force participation had a small increased effect on intentional injuries. Three different variables – occupational risk, high school diploma, and university degree – each provide independent effects when interacting multiplicatively with Indigenous ethnicity; that is, these variables affect only the Indigenous population. Indigenous ethnicity *times* occupation risk increases risk of intentional injury, and that effect is huge, with a change in SRR of 3.96 for each SD increase. Indigenous ethnicity *times* high school diploma decreases risk of intentional injury, and the protective effect is huge, reducing the SRR by 3.19 for each SD increase. Indigenous ethnicity *times* university degree increases risk of intentional injury somewhat, adding 1.246 to SRR for each SD increase.

As shown in Table 7, the best-fitting model predicts that the off-reserve Indigenous population will have SRR of intentional injury 3.98 greater than the total population. That is very close to the observed disparities between the total BC population and the off-reserve Indigenous population (5.18–1 in 1999–2003, and 4.94–1 in 2004–2008) shown in Table 3. The model predicts that the on-reserve Indigenous population will have SRR of intentional injury 4.17 greater than the total BC population. Similarly, that is very close to the observed disparities (5.39–1 in 1999–2003, and 5.28–1 in 2004–2008), reported in Table 3.

**Table 7:** Relative risks predicted by the best-fitting multivariable regression model

X Variable	Total Population	Off-Reserve Indigenous					On-Reserve Indigenous				
	Mean^a^	Mean^a^	Difference^b^	SRR change^c^	L95CL^c^	U95CL^c^	Mean^a^	Difference^b^	SRR change^c^	L95CL^c^	U95CL^c^
Occupation Risk Indigenous	0.046	1.178	1.132	8.60	6.84	10.35	1.135	1.089	8.28	6.59	9.96
Industry Risk Labour Force	0.660	0.760	0.100	0.14	0.07	0.22	0.679	0.019	0.03	0.01	0.04
Employed	0.618	0.590	−0.027	0.10	0.06	0.15	0.473	−0.145	0.55	0.33	0.77
High School Indigenous	0.036	0.658	0.622	−7.07	−9.48	−4.66	0.513	0.477	−5.42	−7.27	−3.57
University Degree Indigenous	0.009	0.066	0.057	2.20	1.40	3.00	0.028	0.019	0.74	0.47	1.01
Total (sum)				3.98					4.17		

^a^population-weighted mean of the x-variable, 2001 and 2006 Census, for the specified population group

^b^difference between mean of the specified population group and mean of the total population

^c^predicted SRR change associated with the difference, calculated as (B x difference), where B is the regression coefficent in the best-fitting multivariable model

Table 7 also shows differences between Indigenous peoples living on- and of-reserve. Those living off-reserve are more likely to complete high school and be employed, both of which are protective against intentional injury, although this is offset by factors that increase risk; that is university education and working in more hazardous occupations.

## Discussion

Intentional injuries resulting in hospitalization are important because of the considerable number, and subsequent individual and societal burdens. The high risk of self-inflicted and inter-personal assaults for Indigenous peoples in Canada has been long recognized [15, 20–26] and is of major concern. The downward trend in risk, and the reduced disparity between populations, shown in our results are both good news. Still, both the overall risk and the disparities remain high, with the risk being approximately three times higher for the Indigenous population for self-inflicted injuries, and four times higher for inter-personal assaults, compared to the total population of BC.

In spite of differing methods to identify the Indigenous populations, our data showing disparity for self-inflicted injuries are consistent with those of Oliver et al. [18], although they found even greater disparity for assaults by another person than did our data. These consistent findings regarding disparity indicate that national and international concern remains warranted [15–24].

Limitations to our data focus on our measurement for injury. We counted hospitalizations due to intentional injury, both for self-inflicted and assaults by another person, but this does not represent the total burden of intentional injuries. Not included in our dataset are intentional injuries that result in mortality, and less severe injuries that do not warrant hospitalization. However, our findings are consistent with previous studies of mortality [34] and primary care utilization [7] due to injuries among the Indigenous population of BC. As well, we included only the “most responsible diagnosis” on the discharge record so that a person who is hospitalized mainly for another diagnosis or an unintentional injury would not be included.

The insurance registry counts about 7% more population than the Census. As a result, the denominators of calculated rates may thus be inflated (resulting in underestimation of the rates), but since this applies to the rates in all population groups, this should not bias the calculated SRRs, which are ratios of two rates. Regarding the off-reserve Indigenous population, the Census counts *more* than the insurance registry, but regarding the on-reserve Indigenous population, the Census counts *less* than the insurance registry. These disparities result from different definitions of “Indigenous”, and inaccuracies of postal codes as a way of identifying Indian reserves. Indigenous population groups within HSDAs among whom we calculated injury risks were not exactly the same as the population groups among whom we ascertained the ecological predictors of risk; however, this mismatch amounts to a bias toward the null hypothesis. Nevertheless, we found strong associations, suggesting that the representativeness of the population groups was not materially affected.

Given the disparity by ethnicity found in this and in our previous work [3, 8], we hypothesized that disparities between the total BC population and the Indigenous populations living off- or on-reserve would be attributable to socioeconomic status, geographic place, and Indigenous ethnicity, or a combination of these factors. Our final multivariable regression model was an excellent fit, but our overall findings were only partially consistent with our hypotheses. The model did not include geographic place, income variables or housing variables.

Intentional injuries were associated with occupational and educational factors. Employment was protective against intentional injury for everyone. Independently of employment, industry risk was associated with increased risk of intentional injury for everyone, and occupational risk was highly influential for both Indigenous population groups. It is possible that industries with high risk for injury are stressful, resulting in self-inflicted injuries or assaults. Further interpretation of these occupational related findings warrants exploration.

Our data show associations between educational attainment and injury risk. Completing high school education provides a buffer for intentional injury for the Indigenous population, while this is not the case for the total BC population. Possibly, this relates to high school completion being the norm for the total BC population (83.4% achievement rate) but less so for Indigenous populations (71.6% for Indigenous off-reserve and 53.0% on-reserve achievement rates). The effect of university education completion increasing the risk of intentional injury for the Indigenous population is paradoxical. It is possible that having a university education sets one apart, leading to both alienation from one’s own culture and racial discrimination in workplaces and elsewhere in which Indigenous peoples are a minority.

Our findings regarding geographic place of residence and specific socioeconomic factors differ across our previous reports on specific categories of injury. We have reported that increased risk corresponded with living in more remote areas for all injuries combined [10], and for the specific categories of unintentional falls [7, 12] and unintentional transportation injuries [14]. The exclusion of geography from our best fitting model for intentional injuries is consistent with the finding on another specific category of injury – iatrogenic injuries [13]. The explanation may relate to the differing mechanisms of injury: in the two latter categories, geography is insignificant when one controls for socioeconomic factors and ethnicity.

When examining differences in injury risk between Indigenous populations living off- and on-reserve, we found large differences for total injuries [8]. Exploring the Indigenous off- and on-reserve population differences for specific categories of injury showed differing patterns and suggests different mechanisms are at play. Both intentional injuries and iatrogenic injuries [11] showed little difference between the Indigenous populations living on-reserve and off-reserve. In contrast, large differences were shown between those two groups for unintentional falls [5, 10] and for unintentional transportation injuries [12]. It is plausible that the risk for unintentional falls and transportation injuries is influenced by the environment, both physical (natural geography, climate) and social (poverty); whereas the risks for intentional injuries and iatrogenic injuries [11] are psychosocial, and involve Indigenous identity.

A question arising from these results is why the time trends of some categories of injury (e.g., unintentional falls, transportation) differ from other categories (e.g., intentional, iatrogenic). The ecological analyses results suggest that it is due to the effect of Indigenous ethnicity. The best-fitting multivariable regression models with unintentional *falls* injury [10] or unintentional *transportation* injury [12] as the outcome have Indigenous ethnicity as a multiplicative interaction with socioeconomic factors. If Indigenous ethnicity remains constant and the socioeconomic disparity diminishes, then the models predict that the injury disparity between the Indigenous and total populations will diminish too. That happened during the period 1991–2010. Therefore, if the goal is to fully close the injury gap in these categories, it would seem prudent to focus efforts on closing the socioeconomic gap.

The best-fitting multivariable regression model with *intentional* injury as the outcome also has Indigenous ethnicity as a multiplicative interaction with socioeconomic factors. However, the benefits of increased high school education (decreased risk of intentional injury) were countered by the effects of increased occupational hazards and increased university education (increased risk of intentional injury). For Indigenous peoples, the trend of socioeconomic improvement had mixed effects on intentional injury risk, therefore the disparity between the Indigenous and total populations did not diminish.

The best-fitting multivariable regression model with *iatrogenic* injuries as the outcome has Indigenous ethnicity and socioeconomic descriptors as independent factors. If Indigenous ethnicity remains constant and the socioeconomic disparities diminish, the model predicts that the injury disparity between the Indigenous and total populations will diminish somewhat, but there will remain a persistent gap due to the independent effect of Indigenous ethnicity. Again, that is consistent with the historical record. Therefore, in order to close the injury gap completely in the future, it would not be sufficient to close the socioeconomic gap. The nature and effect of Indigenous ethnicity in terms of health has to change.

## Conclusions

What does the continuing influence of Indigenous ethnicity on health outcomes actually mean? The history of Canada contains many examples of misguided attempts to modify or eliminate Indigenous ethnicity itself. Numerous reports, including from the United Nations [20, 21], Amnesty International [26] and the Truth and Reconciliation Commission of Canada [25] express major concern for enduring health (including intentional injury) disparities. As the latter report [25] notes, gaps will persist until Canadians address the deeply rooted intense racism, marginalization and poverty endured by Indigenous peoples. We look forward to the day when governments stop fighting against Indigenous peoples [38] and instead, the history, cultural richness and contributions of Indigenous peoples are acknowledged by governments and by the general public so that the physical, psychosocial, and economic conditions for Indigenous peoples can equal those of non-Indigenous Canadians.
